# Risk of cerebrovascular and cardiovascular outcomes in patients with an ICD code for ocular migraine

**DOI:** 10.1038/s41433-026-04479-0

**Published:** 2026-04-29

**Authors:** Jacqueline K. Shaia, Priya Shukla, Hayden Reed, David C. Kaelber, Rishi P. Singh, Katherine E. Talcott

**Affiliations:** 1 Case Western Reserve Department of Population and Quantitative Health Sciences, Cleveland, OH USA; 2 Center for Ophthalmic Bioinformatics, Cole Eye Institute, Cleveland Clinic, Cleveland, OH USA; 3 Cleveland Clinic Lerner College of Medicine of Case Western Reserve University, Cleveland, OH USA; 4 Vanderbilt Eye Institute, Vanderbilt University Medical Center, Nashville, TN USA; 5 Case Western Reserve University School of Medicine, Cleveland, OH USA; 6 Departments of Internal Medicine, Pediatrics, and Population and Quantitative Health Sciences, Case Western Reserve University, Cleveland, OH USA; 7 The Center for Clinical Informatics Research and Education, The MetroHealth System, Cleveland, OH USA; 8 Cleveland Clinic Cole Eye Institute, Cleveland, OH USA; 9 Cleveland Clinic Martin Hospitals, Cleveland Clinic Florida, Stuart, FL USA

**Keywords:** Eye diseases, Epidemiology

## Abstract

**Background:**

It is unknown if ocular migraine patients have an increased risk of cerebrovascular disease. This study aimed to determine if cerebrovascular and cardiovascular events were elevated among those with ocular migraine compared to controls.

**Methods:**

This cohort study utilised a platform of aggregate, de-identified electronic health record data to identify patients with a diagnostic code for ocular migraine based on ICD codes (*n* = 21,949). Ocular migraine patients were compared to ophthalmology (*n* = 1,492,348), migraine with aura (MA) (*n* = 129,099) and migraine without aura (MO) controls. Cohorts were propensity score matched on current age, sex, race/ethnicity, diabetes, essential hypertension, lipoprotein disorders, atherosclerosis and body mass index. Risk ratios and 95% confidence intervals were calculated. A risk ratio between 0.9 and 1.1 was considered not significant.

**Results:**

Compared with ophthalmology controls ≥ 50 years old, patients with a diagnostic code ocular migraine had an increased risk of all outcomes, with a risk ratio of 1.48 (95% CI 1.33, 1.64), transient ischemic stroke 2.68 (2.20, 3.27) and cerebral infarct 1.81 (1.55, 2.10). These findings continued to be significant among the ≥ 65 years old cases and controls. Compared to patients with MA or MO, patients had a comparable risk of all outcomes (*p* > 0.05).

**Conclusion:**

Patients with a diagnostic code for ocular migraine ≥ 50 years old had a 48% increased risk of cerebrovascular and cardiovascular outcomes, compared to ophthalmology controls. Future retrospective and prospective cohort analyses are needed to further verify these findings.

## Introduction

Cerebrovascular and cardiovascular diseases have consistently been in the top 10 leading causes of death within the United States for many years [1–3]. In 2022, 39.5 deaths per 100,000 were attributable to stroke, and 167.2 per 100,000 were attributable to heart disease [2]. Understanding stroke risk factors is vital, as these risks may be modifiable, which would decrease the potential risk for stroke [3]. Several studies have explored an association between stroke and migraine [4–6]. In a 20-year prospective cohort study of over 17,000 women with a migraine diagnosis, strong associations were found between migraine and cerebrovascular/cardiovascular events, such as stroke, even after controlling for confounding factors [4]. Calculated hazard ratios were equal to or above 1.5 for stroke, major cardiovascular disease and angina/coronary revascularisation procedures [7]. A meta-analysis involving over 11,000 records found that risk of haemorrhagic stroke increased by 50% in patients with migraine compared to those without migraine [4]. Another meta-analysis concluded that migraine is associated with an increased risk of ischemic stroke [5]. Overall, this showcases a strong association between stroke and migraine patients [8].

Particularly, this association has been shown to disproportionately impact those with migraine with aura (MA) [8, 9]. A narrative review published in the Journal of Stroke and Cerebrovascular Disease highlighted that the risk of stroke was increased in patients with MA, especially in women younger than 50 years [10]. Further emphasizing this point, a large cohort study of about 240,000 participants from the Taiwan National Health Insurance Research Database also indicated an increased risk of ischemic stroke in migraine patients [6]. Though the highest risk has been shown to be for women younger than 45, smokers and those using oral contraceptives, there were also associations of vascular disease and MA for women older than 45 [11]. These studies highlight that not only is migraine associated with cerebrovascular and cardiovascular events, but ocular symptoms with migraine may hold a higher association.

Although the relationship between cerebrovascular and cardiovascular disease and migraine has been significantly studied, there are no published studies specifically evaluating the risk among patients with a diagnostic code for ocular migraine, defined as meeting the migraine (with or without aura) criteria while having monocular visual disturbances [12]. However, one abstract was published from the Association for Research in Vision and Ophthalmology conference in 2024, where a significant association was found [13].

The purpose of this study was to evaluate whether cerebrovascular and cardiovascular events were elevated among those with ocular migraine compared to other ophthalmology controls. Secondarily, this risk was compared to those with MA and migraine without aura. Results from this study will provide further insight into risk for stroke and other cardiovascular events, with the potential to influence the current standard of care and give providers further information to better treat their patients with a diagnostic code for ocular migraine.

## Methods

This study was conducted utilising the TriNetX platform, a platform that aggregates and de-identifies electronic health record data from 64 healthcare organisations and over 113 million patient records. This data is de-identified, *per the standard defined in Section §164.514(a) of the HIPAA Privacy Rule*. This data has been deemed exempt from ethical approval by the Western Institutional Review Board (IRB) through a qualified expert as defined in Section §164.514(b)(1) of the HIPAA Privacy Rule. All data was collected between June 26th and July 8th, 2024 and obtained through the TriNetX US Collaborative Network.

To identify if ocular migraine patients have an increased risk of cerebrovascular or cardiovascular outcomes, a retrospective cohort study was created and evaluated through propensity score matching. Ocular migraine, MA, MO patients and general ophthalmology controls and outcomes were all identified through using ICD-10 diagnosis codes and CPT procedural codes, which were listed in Supplementary Table 1. ICD-9 codes used for diagnoses occurring prior to 2015 were converted to the corresponding ICD-10 codes based on the Centers for Medicare and Medicaid Services General Equivalence Mappings. Ocular migraine cases in this study were defined as having one or more ICD encounter diagnosis codes of ocular migraine. Cases were compared with three different controls: Cohort 1 - general ophthalmology control, Cohort 2 - MA controls and Cohort 3–MO (migraine without aura) controls through ICD and CPT coding (Supplementary Table 1). Patients with any ICD encounter diagnosis of photopsia (H53.19) and visual disturbances (H53) were excluded to best identify true ocular migraine patients. Outcomes included cerebral infarct, transient cerebral ischemic attack, myocardial infarction and all outcomes used together (Supplementary Table 1). A negative control of allergic dermatitis was also utilised to assist with validating this analysis. Any patient who experienced an outcome of interest prior to meeting the inclusion criteria was excluded from this analysis.

As cerebrovascular and cardiovascular outcomes are associated with age, with most strokes occurring after the age of 50 [14], this analysis was conducted twice at two different age groups: 50 years or older and 65 years or older. In addition, to be included in the study, patients had to meet the required inclusion criteria on or before 12/31/2019 to allow for a minimum of 5 years of follow-up time.

A propensity score analysis utilising a greedy 1:1 matching algorithm built into the TriNetX analytics platform was applied for this study. Patients were matched on potential confounding factors including current age, sex, race/ethnicity (white, Black, Hispanic/Latino), diabetes, essential hypertension, lipoprotein disorders, atherosclerosis and body mass index (defined as a BMI at least 1 day prior to meeting the index event and categorically defined as < 25 kg/m^2^, 25 < 30 kg/m^2^ and 30+ kg/m^2^). After matching, any standard difference less than 0.1 was considered a successful match. The risk ratios of all the outcomes and 95% confidence intervals were calculated for this study. To avoid overinterpreting small effect sizes, a risk ratio between 0.9 and 1.1 was considered not significant. All analyses were conducted through the TriNetX platform and R Studio. All pertinent ICD codes were included in Supplementary Table 1. We followed the STOBE guidelines for observational cohort studies in the design and write-up of this study [15].

## Results

**Fig. 1:**
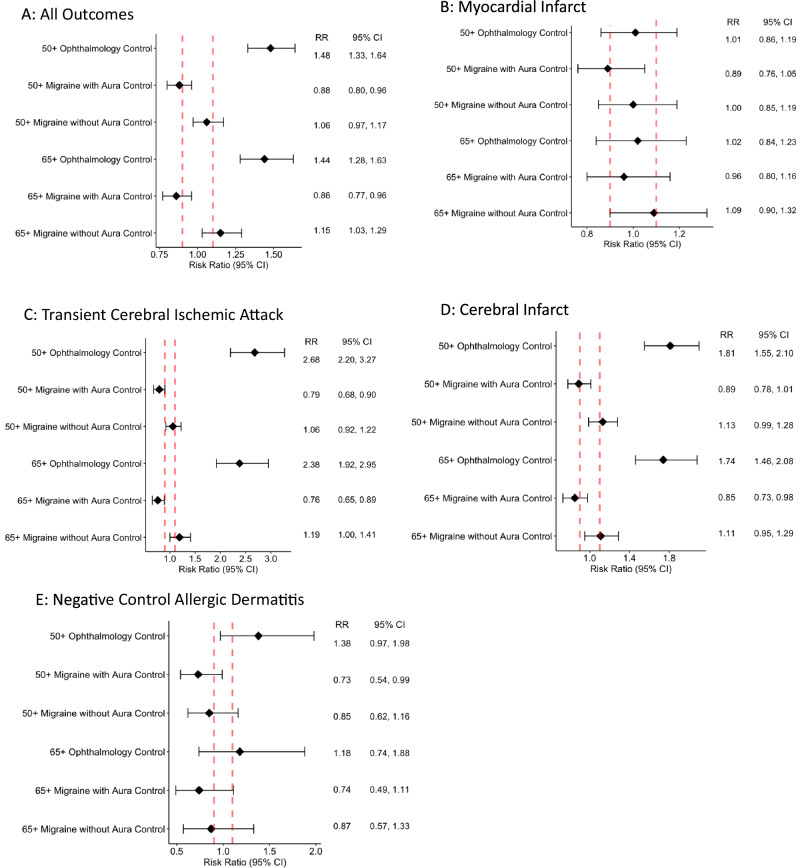
Risk of Cardiovascular Outcomes in Ocular Migraine Patients Compared to Controls. **A**–**E** Risk of Cardiovascular Outcomes in Ocular Migraine Patients Compared to Controls. Forest Plots All Outcomes.

Among those 50 years or older, there were 21,949 patients coded for ocular migraine within TriNetX and 1,492,348 coded as ophthalmology controls. Cohort 1 compared individuals 50 years or older with an ICD code for ocular migraine to ophthalmology controls. After propensity score matching, patients had a mean age of 66.6 years (SD 11.1) with 67% being female. 71% of patients were white, and 6.4% identified as having a Black race. Overall, propensity matching was successful for all variables with standard differences < 0.1 (Table 1). Compared to ophthalmology controls, ICD coded ocular migraine patients had an increased risk of all outcomes with a risk ratio of 1.48 (95% CI 1.33, 1.64) (Fig. 1A). When stratified by outcome, patients with a diagnostic code for ocular migraine had an increased risk of transient cerebral ischemic attacks at 2.68 (2.20, 3.27) and cerebral infarcts at 1.81 (1.55, 2.10) (Fig. 1C, D). Patients with a diagnostic code for was comparable to ophthalmology controls for myocardial infarctions with a risk ratio of 1.01 (0.86, 1.19) (Fig. 1B).

**Table 1:** Age 50+ Ocular Migraine Compared to Ophthalmology Controls.

	Before Matching *N* (%)			After Matching *N* (%)		
	Ocular Migraine (*n* = 21,949)	Ophthalmology Control (*n* = 1,492,348)	SMD	Ocular Migraine (*n* = 17,737)	Ophthalmology Control (*n* = 17,737)	SMD
Demographics						
Current Age mean (SD)	66.6 (11.1)	71.3 (11.5)	0.43	66.6 (11.1)	66.6 (11.1)	0.001
Race/Ethnicity ^a^						
White (%)	12,614 (71.1)	815, 278 (63.1)	0.17	12,614 (71.1)	12,636 (71.2)	0.003
Black (%)	1140 (6.4)	189,056 (14.6)	0.27	1140 (6.4)	1143 (6.4)	0.001
Hispanic or Latino (%)	703 (4.0)	97,581 (7.5)	0.15	703 (4.0)	697 (3.9)	0.002
Female Sex (%)	12,000 (67.7)	709,011 (54.9)	0.27	12,000 (67.7)	12,005 (67.7)	0.001
Comorbidities						
Diabetes (E08–E13) (%)	1486 (8.4)	261,087 (20.2)	0.34	1486 (8.4)	1487 (8.4)	<0.001
Essential Hypertension (I10) (%)	4839 (27.3)	431,396 (33.4)	0.13	4839 (27.3)	4831 (27.2)	0.001
Lipidemia Disorders (E78) (%)	4947 (27.9)	385,497 (29.8)	0.04	4947 (27.9)	4945 (27.9)	<0.001
Atherosclerosis (I70) (%)	297 (1.7)	29,118 (2.3)	0.04	297 (1.7)	276 (1.6)	0.009
Tobacco Use (Z72.0) (%)	136 (0.8)	16,692 (1.3)	0.05	136 (0.8)	117 (0.7)	0.01
Body Mass Index (BMI)						
BMI mean (SD)	28.8 (6.9)	29.5 (7.0)	0.11	28.8 (6.9)	28.8 (6.9)	0.006
0–24.9 kg/m2	3235 (18.2)	148,760 (11.5)	0.19	3235 (18.2)	3230 (18.2)	0.001
25 < 30 kg/m2	3799 (21.4)	198,581 (15.4)	0.16	3799 (21.4)	3801 (21.4)	<0.001
30 + kg/m2	3470 (19.6)	202,394 (15.7	0.10	3470 (19.6)	3468 (21.5)	<0.001

*SMD* standardised mean difference, *SD* standard deviation.

^a^ race and ethnicity were determined based on presence of these designations within the electronic health record.

This analysis was run a second time for individual aged 65 years or older. Again, matching was deemed successful with all values having a standard difference of < 0.01 (Supplementary Table 2). These results were comparable to those of the 50 years + cohort where patients with a diagnostic code for ocular migraine had an increased risk of all outcomes at 1.44 (1.28, 1.63) (Fig. 1A), transient cerebral ischemic attacks at 2.38 (1.92, 2.95) (Fig. 1C) and cerebral infarcts at 1.74 (1.46, 2.08) (Fig. 1D). Overall, ICD coded ocular migraine patients had significantly increased risk of transient cerebral ischemic attacks and cerebral infarcts compared to ophthalmology controls (*p* < 0.05) (Fig. 1C, D).

Cohort 2 compared ICD-coded ocular migraine patients to patients with MA. For individuals 50 years and older, the average age after propensity matching was 66 (SD 11), with 66% identifying as female. Propensity matching continued to be successful with all comorbidities having a standard difference < 0.1 (Table 2). With all outcomes combined, patients with a diagnostic code for ocular migraine had a comparable risk to MA patients with a risk ratio of 0.88 (0.80, 0.96) (Fig. 1A). Even after stratifying by the different outcomes, ICD coded ocular migraine patients and ICD coded MA patients continued to have comparable risk (*p* > 0.05) (Fig. 1B–D).

**Table 2:** Age 50 + Ocular Migraine Compared to Migraine with Aura Controls.

	Before Matching *N* (%)			After Matching *N* (%)		
	Ocular Migraine (*n* = 21,949)	Migraine with Aura Control (*n* = 129,099)	SMD	Ocular Migraine (*n* = 17,737)	Migraine with Aura Control (*n* = 17,737)	SMD
Demographics						
Current Age mean (SD)	66.6 (11.1)	63.8 (10.1)	0.26	66.6 (11.1)	66.5 (11.0)	0.008
Race/Ethnicity ^a^						
White	12,614 (71.1)	87,856 (73.5)	0.05	12,614 (71.1)	12,685 (71.5)	0.009
Black	1140 (6.4)	8095(6.8)	0.01	1140 (6.4)	1084 (6.1)	0.01
Hispanic or Latino	703 (4.0)	6257 (5.2)	0.06	703 (4.0)	630 (3.6)	0.02
Female Sex	12,000 (67.7)	89,342 (74.7)	0.16	12,000 (67.7)	11,980 (67.5)	0.002
Comorbidities						
Diabetes (E08–E13)	1486 (8.4)	10,092 (8.4)	0.002	1486 (8.4)	1361 (7.7)	0.03
Essential Hypertension (I10)	4839 (27.3)	31,601 (26.4)	0.02	4839 (27.3)	4736 (26.7)	0.01
Lipidemia Disorders (E78)	4947 (27.9)	32,313 (27.0)	0.02	4947 (27.9)	4888 (27.6)	0.007
Atherosclerosis (I70)	297 (1.7)	1662 (1.4)	0.02	297 (1.7)	216 (1.2)	0.04
Tobacco Use (Z72.0)	136 (0.8)	1966 (1.6)	0.08	136 (0.8)	101 (0.6)	0.02
Body Mass Index (BMI)						
BMI mean (SD)	28.8 (6.9)	29.1 (6.9)	0.05	28.8 (6.9)	28.8 (6.8)	0.004
0–24.9 kg/m2	3235 (18.2)	21,919 (18.3)	0.002	3235 (18.2)	3210 (18.1)	0.004
25 < 30 kg/m2	3799 (21.4)	25,853 (21.6)	0.005	3799 (21.4)	3774 (21.3)	0.003
30 + kg/m2	3470 (19.6)	24,912 (20.8)	0.03	3470 (19.6)	3456 (19.5)	0.002

*SMD* standardised mean difference, *SD* standard deviation.

^a^race and ethnicity were determined based on the presence of these designations within the electronic health record.

Cohort 2 was run a second time among those aged 65 and older. After determining this was a successful propensity match in Supplementary Table 3, the risk ratio of all combined outcomes was again comparable between both ICD coded ocular migraine patients and ICD coded MA patients with a risk of 0.86 (0.77, 0.96) (Fig. 1A). Even after stratifying by type of outcome, the ICD coded ocular migraine and MA groups continued to be comparable (*p* > 0.05) (Fig. 1B–D).

Cohort 3 compared ICD-coded patients with ocular migraine to patients with MO. Propensity matching was deemed successful with those aged 50 and over. The average age of the cohort was 66, and 67% were female (Table 3). After matching, ICD coded ocular migraine patients had comparable risk of all outcomes to those with MO with a risk of 1.06 (0.97, 1.17) (Fig. 1A). When stratifying by outcome type, there was no difference between groups (*p* > 0.05) meaning patients with a diagnostic code for ocular migraine had comparable risk to those with MO for all outcomes (Fig. 1B–D).

**Table 3:** Age 50 + Migraine without Aura Control.

	Before Matching *N* (%)			After Matching *N* (%)		
	Ocular Migraine (*n* = 21,949)	Migraine without Aura Control (*n* = 171,479)	SMD	Ocular Migraine (*n* = 17,736)	Migraine without Aura Control (*n* = 17,736)	SMD
Demographics						
Current Age mean (SD)	66.6 (11.1)	62.4 (9.2)	0.41	66.6 (11.1)	66.5 (11.0)	0.007
Race/Ethnicity ^a^						
White	12,614 (71.1)	107,885 (69.5)	0.04	12,613(71.1)	12,657 (71.4)	0.005
Black	1140 (6.4)	13,789 (8.9)	0.09	1140 (6.4)	1108 (6.2)	0.007
Hispanic or Latino	703 (4.0)	9180 (5.9)	0.09	703 (4.0)	662 (3.7)	0.012
Female Sex	12,000 (67.7)	120,535 (77.6)	0.23	12,000 (67.7)	11,983 (67.6)	0.002
Comorbidities						
Diabetes (E08–E13)	1486 (8.4)	13,856 (8.9)	0.02	1486 (8.4)	1387 (7.8)	0.02
Essential Hypertension (I10)	4839 (27.3)	40,060(25.8)	0.03	4839 (27.3)	4735 (26.7)	0.013
Lipidemia Disorders (E78)	4947 (27.9)	40,580 (26.1)	0.04	4947 (27.9)	4920 (27.7)	0.003
Atherosclerosis (I70)	297 (1.7)	1740 (1.1)	0.05	296 (1.7)	218 (1.2)	0.04
Tobacco Use (Z72.0)	136 (0.8)	2992 (1.9)	0.10	136 (0.8)	108 (0.6)	0.02
Body Mass Index (BMI)						
BMI mean (SD)	28.8 (6.9)	29.4 (7.2)	0.08	28.8 (6.9)	28.9 (7.0)	0.009
0–24.9 kg/m2	3235 (18.2)	28,394 (18.3)	0.008	3235 (18.2)	3242 (18.3)	0.001
25 < 30 kg/m2	3799 (21.4)	33,421 (21.5)	0.002	3799 (21.4)	3747 (21.1)	0.007
30 + kg/m2	3470 (19.6)	34247 (21.1)	0.05	3470 (19.6)	3449 (19.4)	0.003

*SMD* standardised mean difference, *SD* standard deviation.

^a^ race and ethnicity were determined based on the presence of these designations within the electronic health record.

Cohort 3 was run among those aged 65 and older, and propensity matching was successful (Supplementary Table 4). When observing risk of all outcomes, ocular migraine patients had a risk of 1.15 (1.03, 1.29), which was deemed comparable to those with MO (Fig. 1A). When observing each outcome separately, patients with a diagnostic code for ocular migraine continued to have comparable risk to those with ICD coded MO (*p* > 0.05) (Fig. 1B–D).

Allergic dermatitis was the negative control for this study and showed no difference between groups for all cohort comparisons (Fig. 1E).

## Discussion

Our study evaluated the risk of cerebrovascular and cardiovascular events among those with an ICD code diagnosis of ocular migraine. Compared to general ophthalmology controls, patients with a diagnostic code for ocular migraine had a 48% increased risk of any cerebrovascular event. This was especially increased for transient cerebral ischemic attacks, where ocular migraine patients had a 2.68 times increased risk compared to ophthalmology controls. Cerebral infarcts had an 81% increased risk among ocular migraine patients compared to ophthalmology controls. Those with an ocular migraine diagnostic code were found to have a comparable risk to those with a migraine diagnostic code, regardless of aura status (*p *> 0.05).

Overall, our study supports other study findings in that patients with an ICD code for ocular migraine have increased risks of cerebral ischemia and transient ischemic attacks [5, 9, 16]. There are several possible mechanisms that may explain the association between stroke and migraine. First, patients with migraine may have a higher risk profile for cardiovascular conditions compared to controls [16]. This includes migraine patients having a higher blood pressure and increased cardiac disease risk according to the Framingham Risk Score [17]. Interestingly, MO patients did not have an increased risk of a Framingham Risk Score [17], showcasing that MO patients do not appear to have the same cardiovascular risk profile as MA patients [16, 17]. Future studies may want to observe if a difference in cardiovascular risk profile is present among ocular migraine patients. Additionally, migraine patients have been found to have higher insulin resistance [18] and dyslipidemia [17] compared to controls. This places them at a higher risk for metabolic syndrome and thus increases their risk for cardiovascular disease [19].

Other suggested mechanisms include that migraine patients have a decreased threshold for cortical spreading depression, making them more susceptible to ischemic brain injury [9, 16, 20]. This mechanism has also been suggested to affect the retina in those with ocular migraines [21]. Altered endothelial cells may also contribute to this increased risk of stroke in migraine patients; however, this is still debated [9, 22]. This may play a role specifically for ocular migraine, where vasospasms have been noted in the retinal vessels of these patients [21]. More research is needed to better understand this relationship and determine if migraine subtypes, including MA and ocular migraine, are at an increased risk of stroke compared to those without visual symptoms [12].

Further, it should be acknowledged that this study depends on the validity and accuracy of ICD coding for ocular migraine, which could explain the results of this study. Through this analysis, we aimed to evaluate if patients with an ICD code for ocular migraine had increased risk of cerebrovascular and cardiovascular events and based on our findings, we determined that this area of research necessitates further study and exploration. Future studies should include granular retrospective cohort and prospective cohort evaluations.

There were several limitations to this study. First, our analysis solely used ICD coding, which has inherent limitations as described above. In addition, several reports indicate that an earlier age of onset of migraine might be a predictive factor of stroke [6, 11]. We were not able to evaluate the specific age at onset, as the data platform does not allow for the identification of a diagnosis age group and then later determine the risk of stroke development. We also did not obtain medication histories in this analysis, and this platform is not able to capture medical adherence. Lastly, we aimed to control for many vital confounders; however, unmeasured variables not available in TriNetX, including socioeconomic status, could not be controlled for.

Within this population, patients with a diagnostic code for ocular migraines appear to be associated with an increased risk of ischemic stroke and cerebral infarct compared to ophthalmology controls. This analysis showcased comparable risk to that of migraine patients. More research is needed to elucidate the mechanism of this relationship, such as cohort analyses, to further verify these findings.

## Summary

### What is known about this topic

- Although the relationship between cerebrovascular and cardiovascular disease and migraine has been significantly studied, there are no published studies specifically evaluating the risk among patients with ocular migraine, defined as meeting the migraine (with or without aura) criteria while having monocular visual disturbances.

### What this study adds

- Ocular migraine patients ≥ 50 years old have a 48% increased risk of cerebrovascular and cardiovascular outcomes, including ischemic stroke and cerebral infarct, compared to ophthalmology controls. This risk is comparable to those with MA or MO.

## Supplementary information

Supplemental Materials

## Data Availability

The data used in this analysis can be accessed through the TriNetX platform.
