# Supplementary material for: Risk of cerebrovascular and cardiovascular outcomes in patients with an ICD code for ocular migraine
Source: Eye (Lond). 2026 Apr 29;40(10):1515–20. doi: 10.1038/s41433-026-04479-0 (PMC13341769; doi:10.1038/s41433-026-04479-0)
Supplement: Supplementary file 1 — Supplemental Materials [file 41433_2026_4479_MOESM1_ESM.docx]

**Supplemental Tables**

Supplemental Table 1: ICD codes utilized in analysis

| Condition | ICD Code |
| --- | --- |
| **Cohorts** | |
| Ocular Migraine | G43.B  (excluding H53.19 photopsia, H53 Visual Disturbances) |
| Ophthalmology Controls | Encounter for examination of eyes and vision (CPT Z01.0) **or** Ophthalmology services and procedures (CPT code 1012793) |
| Migraine (all) | G43 |
| Migraine with Aura | G43.1 |
| Migraine without Aura | G43.0 |
| **Outcomes** | |
| Cerebral Infarction I63 | I63 |
| Transient Cerebral Ischemic Attach | G45.9 |
| Myocardial Infarction | I21 |
| Negative Control: allergic dermatitis | L23.7 |
| **Propensity Matching** | |
| Diabetes | E08-E13 |
| Essential Hypertension | I10 |
| Disorders of Lipoprotein metabolism and other | E78 |
| Atherosclerosis | I70 |

**Supplemental Table 2: Age 65+ Ocular Migraine Compared to Ophthalmology Controls.**

|  | Before Matching  N (%) | | | | After Matching  N (%) | | |  |  |  |  |
| --- | --- | --- | --- | --- | --- | --- | --- | --- | --- | --- | --- |
|  | Ocular Migraine  (n = **11,770**) | Ophthalmology Control  (n = **1,048,626**) | | SMD | Ocular Migraine  (n = **9,314**) | Ophthalmology Control  (n = **9,314**) | SMD |  |  |  |  |
| Demographics |  |  |  | |  |  |  |  |  |  |  |
| Current Age mean (SD) | 75.3 (7.6) | 77.3 (8.0) | 0.26 | | 75.3 (7.5) | 75.3 (7.6) | 0.003 |  |  |  |  |
| Race/Ethnicity ^a^ |  |  |  | |  |  |  |  |  |  |  |
| White | 6,929 (74.4) | 589,724 (65.8) | 0.19 | | 6,929 (74.4) | 6,951 (74.6) | 0.005 |  |  |  |  |
| Black | 467 (5.0) | 120,239 (13.4) | 0.29 | | 467 (5.0) | 472 (5.1) | 0.002 |  |  |  |  |
| Hispanic or Latino | 242 (2.6) | 56,491 (6.3) | 0.18 | | 242 (2.6) | 240 (2.6) | 0.001 |  |  |  |  |
| Female Sex | 6,078 (65.3) | 499,614 (55.8) | 0.20 | | 6,078 (65.3) | 6,095 (65.4) | 0.005 |  |  |  |  |
| Comorbidities |  |  |  | |  |  |  |  |  |  |  |
| Diabetes (E08-E13) | 1,021 (11.0) | 190,777 (21.3) | 0.28 | | 1,021 (11.0) | 1,036 (11.1) | 0.005 |  |  |  |  |
| Essential Hypertension (I10) | 3,368 (36.2) | 337,313 (37.7) | 0.03 | | 3,368 (36.2) | 3,364 (36.1) | 0.001 |  |  |  |  |
| Lipidemia Disorders (E78) | 3,529 (37.9) | 299,674 (33.5) | 0.09 | | 3,529 (37.9) | 3,520 (37.8) | 0.002 |  |  |  |  |
| Atherosclerosis (I70) | 260 (2.8) | 25,965 (2.9) | 0.006 | | 260 (2.8) | 230 (2.5) | 0.02 |  |  |  |  |
| Tobacco Use (Z72.0) | 61 (0.7) | 9,450 (1.1) | 0.04 | | 61 (0.7) | 45 (0.5) | 0.02 |  |  |  |  |
| Body Mass Index (BMI) | | | | | | | |  |  |  | Body Mass Index (BMI) |
| BMI mean (SD) | 28.2 (6.2) | 29.2 (6.7) | 0.15 | | 28.2 (6.2) | 28.4 (6.5) | 0.04 |  |  |  |  |
| 0-24.9 kg/m2 | 1,723 (18.5) | 102,948 (11.5) | 0.20 | | 1,723 (18.5) | 1,716 (18.4) | 0.002 |  |  |  |  |
| 25 <30 kg/m2 | 2,139 (23.0) | 139,273 (15.5) | 0.19 | | 2,139 (23.0) | 2,139 (23.0) | <0.001 |  |  |  |  |
| 30 + kg/m2 | 1,737 (18.6) | 133,842 (14.9) | 0.10 | | 1,737 (18.6) | 1,738 (18.7) | <0.001 |  |  |  |  |
| Abbreviations: SMD standardized mean difference, SD standard deviation  ^a^ race and ethnicity were determined based on presence of these designations within the electronic health record | | | | | | | |  |  |  |  |

**Supplemental Table 3 – Age 65+ Migraine with Aura Control.**

|  | Before Matching  N (%) | | | After Matching  N (%) | | |
| --- | --- | --- | --- | --- | --- | --- |
|  | Ocular Migraine  (n = 11,770) | Migraine with Aura Control  (n = 55,141) | SMD | Ocular Migraine  (n = 9,314) | Migraine with Aura Control  (n = 9,314) | SMD |
| Demographics |  |  |  |  |  |  |
| Current Age mean (SD) | 75.3 (7.6) | 73.7 (6.9) | 0.22 | 75.3 (7.6) | 75.2 (7.6) | 0.005 |
| Race/Ethnicity ^a^ |  |  |  |  |  |  |
| White | 6,929 (74.4) | 38,914 (77.5) | 0.07 | 6,929 (74.4) | 6,982 (75.0) | 0.01 |
| Black | 467 (5.0) | 2,326 (4.6) | 0.02 | 467 (5.0) | 443 (4.8) | 0.01 |
| Hispanic or Latino | 242 (2.6) | 1,580 (3.1) | 0.03 | 242 (2.6) | 207 (2.2) | 0.03 |
| Female Sex | 6,078 (65.3) | 35,759 (71.2) | 0.13 | 6,078 (65.3) | 6,042 (64.9) | 0.008 |
| Comorbidities |  |  |  |  |  |  |
| Diabetes (E08-E13) | 1,021 (11.0) | 5,412 (10.8) | 0.006 | 1,021 (11.0) | 948 (10.2) | 0.03 |
| Essential Hypertension (I10) | 3,368 (36.2) | 17,842 (35.5) | 0.01 | 3,368 (36.2) | 3,337 (35.8) | 0.007 |
| Lipidemia Disorders (E78) | 3,529 (37.9) | 18,844 (37.5) | 0.007 | 3,529 (37.9) | 3,522 (37.8) | 0.002 |
| Atherosclerosis (I70) | 260 (2.8) | 1,282 (2.6) | 0.02 | 260 (2.8) | 210 (2.3) | 0.03 |
| Tobacco Use (Z72.0) | 61 (0.7) | 593 (1.2) | 0.06 | 61 (0.7) | 52 (0.6) | 0.01 |
| Body Mass Index (BMI) | | | | | | |
| BMI mean (SD) | 28.2 (6.2) | 28.3 (6.2) | 0.006 | 28.2 (6.2) | 28.3 (6.1) | 0.01 |
| 0-24.9 kg/m2 | 1,723 (18.5) | 9,932 (19.8) | 0.03 | 1,723 (18.5) | 1,708 (18.3) | 0.004 |
| 25 <30 kg/m2 | 2,139 (23.0) | 11,631 (23.2) | 0.005 | 2,139 (23.0) | 2,122 (22.8) | 0.004 |
| 30 + kg/m2 | 1,737 (18.6) | 9,522 (19.0) | 0.008 | 1,737 (18.6) | 1,699 (18.2) | 0.01 |
| Abbreviations: SMD standardized mean difference, SD standard deviation  ^a^ race and ethnicity were determined based on presence of these designations within the electronic health record | | | | | | |

|  | Before Matching  N (%) | | | After Matching  N (%) | | |
| --- | --- | --- | --- | --- | --- | --- |
|  | Ocular Migraine  (n = **11,770**) | Migraine without Aura Control  (n = 64,975) | SMD | Ocular Migraine  (n = **9,314**) | Migraine without Aura Control  (n = **9,314**) | SMD |
| Demographics | | | | | | |
| Current Age mean (SD) | 75.3 (7.6) | 72.5 (6.4) | 0.39 | 75.3 (7.6) | 75.2 (7.5) | 0.008 |
| Race/Ethnicity ^a^ | | | | | | |
| White | 6,929 (74.4) | 41,450 (73.0) | 0.03 | 6,929 (74.4) | 7,002 (75.2) | 0.02 |
| Black | 467 (5.0) | 3,709 (6.5) | 0.07 | 467 (5.0) | 442 (4.7) | 0.01 |
| Hispanic or Latino | 242 (2.6) | 2,257 (4.0) | 0.08 | 242 (2.6) | 217 (2.3) | 0.02 |
| Female Sex | 6,078 (65.3) | 42,967 (75.7) | 0.23 | 6,078 (65.3) | 6,077 (65.2) | <0.001 |
| Comorbidities |  |  |  |  |  |  |
| Diabetes (E08-E13) | 1,021 (11.0) | 6,876 (12.1) | 0.04 | 1,021 (11.0) | 963 (10.3) | 0.02 |
| Essential Hypertension (I10) | 3,368 (36.2) | 19,800 (34.9) | 0.03 | 3,368 (36.2) | 3,343 (35.9) | 0.006 |
| Lipidemia Disorders (E78) | 3,529 (37.9) | 20,918 (36.9) | 0.02 | 3,529 (37.9) | 3,506 (37.6) | 0.005 |
| Atherosclerosis (I70) | 260 (2.8) | 1,224 (2.2) | 0.04 | 260 (2.8) | 190 (2.0) | 0.05 |
| Tobacco Use (Z72.0) | 61 (0.7) | 855 (1.5) | 0.08 | 61 (0.7) | 47 (0.5) | 0.02 |
| Body Mass Index (BMI) | | | | | | |
| BMI mean (SD) | 28.2 (6.2) | 28.5 (6.6) | 0.05 | 28.2 (6.2) | 28.2 (6.3) | 0.004 |
| 0-24.9 kg/m2 | 1,723 (18.5) | 11,113 (19.6) | 0.03 | 1,723 (18.5) | 1,691 (18.2) | 0.009 |
| 25 <30 kg/m2 | 2,139 (23.0) | 12,871 (22.7) | 0.007 | 2,139 (23.0) | 2,120 (22.8) | 0.005 |
| 30 + kg/m2 | 1,737 (18.6) | 11,471 (20.2) | 0.04 | 1,737 (18.6) | 1,752 (18.8) | 0.004 |
| Abbreviations: SMD standardized mean difference, SD standard deviation  ^a^ race and ethnicity were determined based on presence of these designations within the electronic health record | | | | | | |

**Supplemental Table 4: Age 65+ Migraine without Aura Control**
